# Relapse risk, frustration tolerance, and motivational readiness for change in substance use disorders

**DOI:** 10.1186/s40359-025-03560-9

**Published:** 2025-12-01

**Authors:** Ahmed Zaher, Ghada Mourad, Fatma Ibrahim

**Affiliations:** Department of Psychiatric and Mental Health Nursing, Faculty of Nursing, Ain Shams University, Cairo, 11517 Egypt

**Keywords:** Substance use disorders, Relapse risk, Frustration tolerance, Motivational readiness for change, Addiction recovery

## Abstract

**Background:**

Substance use disorders (SUDs) are chronic brain diseases characterized by compulsive use of legal or illegal substances despite harm. Although pharmacological and psychosocial interventions have advanced, relapse remains common. Understanding psychological factors such as frustration tolerance and readiness to change is essential for guiding culturally sensitive relapse‑prevention strategies.

**Methods:**

A cross-sectional study was conducted among 288 Egyptian outpatients diagnosed with substance use disorders, predominantly involving opioids and hallucinogens, with most participants reporting polysubstance use (two to four substances). Validated tools assessed relapse risk (Stimulant Relapse Risk Scale), frustration tolerance (Frustration Discomfort Scale) and motivational readiness for change (Stages of Change Readiness and Treatment Eagerness Scale). Descriptive statistics and Pearson correlations examined associations among these constructs and socio‑demographic and clinical factors.

**Results:**

High relapse risk correlated with emotionality issues (62.8%) and lack of negative expectancies (64.2%). Frustration tolerance was low, with entitlement (61.8%) and achievement (64.6%) most affected. Motivational readiness showed significant barriers, with ambivalence prevalent in high‑risk patients (49.3%). Relapse risk positively correlated with frustration intolerance (*r* = 0.482, 95% CI 0.39–0.57, *p* < 0.001) and negatively correlated with motivational readiness (*r* = − 0.399, 95% CI − 0.49 to − 0.30, *p* < 0.001). Motivational readiness inversely correlated with frustration intolerance (*r* = − 0.643, 95% CI − 0.71 to − 0.57, *p* < 0.001).

**Conclusions:**

Emotional dysregulation, low frustration tolerance and ambivalence are common among Egyptian patients with SUDs. Tailored interventions focusing on emotion regulation, enhancing frustration tolerance and strengthening motivation may support recovery and reduce relapse risk.

## Introduction

Substance use disorders (SUDs) are chronic, relapsing brain diseases characterized by compulsive drug taking despite harmful consequences. The World Health Organization classifies SUDs into alcohol‑use and drug‑use disorders; drug‑use disorders include opioid‑, cocaine‑, amphetamine‑, cannabis‑ and other drug‑use disorders [1]. The risk and speed of developing an addiction vary across substances: opioids and heroin have particularly high addiction liability and can induce dependence rapidly [2].

Globally, SUDs impose a major burden on morbidity, mortality and economics. For example, the age‑standardized disability‑adjusted life‑year (DALY) rate for SUDs in the Middle East and North Africa (MENA) increased from 190.1 to 234.93 per 100 000 people between 1990 and 2019—a 23% rise [3]. Opioids account for the largest share of drug‑related DALYs in many MENA countries [4].

People with SUDs frequently experience medical and psychiatric comorbidities. The National Institute on Drug Abuse reports high rates of co‑occurring mental disorders (depression, anxiety) and infections such as HIV and hepatitis C, as well as chronic pain and cardiovascular disease [5]. These comorbidities complicate treatment and must be addressed concurrently [6]. Beyond individual health, SUDs carry a heavy social burden: stigma, discrimination, unemployment and family pressures can undermine recovery [7]. In Egypt, strong familial expectations, limited access to specialized rehabilitation and economic hardship amplify relapse vulnerability [8].

Relapse the return to substance use after a period of abstinence is common. Recent statistics suggest that 40–60% of individuals with addiction relapse; more than 85% relapse within one year of treatment [9]. Relapse rates vary by substance: nicotine, heroin and alcohol have one‑year relapse rates of 80–95%, whereas stimulant users relapse at approximately 50% in the first year [10]; about half of marijuana users relapse within 24 h after re‑exposure [11]. These differences highlight the importance of identifying the primary substance used, yet many patients, including those in this study, are polysubstance users. Addiction risk also differs by drug; opioid painkillers and heroin carry higher addiction liability [12].

Theoretical models attribute relapse to a combination of emotional, cognitive and behavioral vulnerabilities [13]. Emotional dysregulation characterized by heightened anxiety, negative affect and difficulty coping has been identified as a critical driver of relapse [14]. Frustration tolerance, defined as the capacity to withstand distress and setbacks without resorting to substance use, remains an understudied yet potentially modifiable factor in the context of addiction [15]. Low frustration tolerance has been consistently linked to impulsivity, emotional instability, and heightened stress vulnerability, mechanisms that in turn may amplify relapse risk and hinder long-term recovery [16].

Motivational readiness for change, often operationalized through the Transtheoretical Model stages (precontemplation, contemplation, preparation, action and maintenance), reflects an individual’s willingness and preparedness to alter substance‑related behaviors [17]. Ambivalence or low motivation can hinder treatment engagement and increase the likelihood of relapse. These psychological constructs may interact: frustration intolerance can erode motivation by amplifying negative affect, whereas low motivation may exacerbate stress responses, creating a vicious cycle [18, 19].

Empirical studies on frustration tolerance and motivational readiness have been conducted in Western contexts, but there is limited research among Egyptian populations. Cultural norms, stigma and socio‑economic stressors may shape how these constructs manifest. This study therefore seeks to address this gap and inform culturally adapted relapse‑prevention strategies.

### Aim

The present study aimed to examine psychological factors associated with relapse risk among Egyptian patients with substance use disorders (SUDs). The specific objectives were to:

1. Assess levels of relapse risk across multiple domains (e.g., emotionality, compulsivity, expectancies) in an outpatient clinical sample.
2. Evaluate frustration tolerance and its subdimensions (emotional intolerance, entitlement, discomfort intolerance, achievement) as potential vulnerability factors for relapse.
3. Investigate motivational readiness for change, including recognition, ambivalence, and behavioral steps, as protective or risk factors.
4. Explore the interrelationships among relapse risk, frustration tolerance, and motivational readiness within the sociocultural context of Egypt.
5. Inform the development of culturally adapted relapse-prevention strategies tailored to Egyptian patients, extending the evidence base beyond predominantly Western research.

## Methods

### Study design

We employed a descriptive correlational cross‑sectional design, reported according to the STROBE guidelines.

### Study setting

The study was conducted at the outpatient addiction clinics of El-Abasia Mental Health Hospital, which serves nearly 100,000 patients annually, including 1,300 inpatients across 46 specialized units. The outpatient clinic provides free, comprehensive care for mental illness and addiction through reception areas, consultation rooms, group therapy spaces, and education and follow-up facilities. Treatment includes thorough psychiatric assessment, individualized counseling focused on relapse prevention, cognitive-behavioral therapy to address coping skills, triggers, and cognitive restructuring. Patients also receive group therapy that integrates psychoeducation, peer support, and coping strategies, with medication-assisted treatment (such as methadone or buprenorphine) offered when clinically indicated. Ongoing after-care and family counseling further support recovery.

### Participants and sample size

A convenience sample of 288 patients with substance use disorders was recruited from outpatient addiction clinics. The sample size was calculated using Thompson [20] formula, based on an annual outpatient census of 1,156 patients, with a 95% confidence level and a 5% margin of error to ensure adequate statistical power for the study. Missing data were minimal (< 2%) and handled through pairwise deletion. The inclusion criteria required participants to be diagnosed by a psychiatrist with a substance use disorder, without co-morbidities, according to DSM-5. Participants also needed to be able to communicate coherently and relevantly. Patients with drug-induced psychosis were excluded from the study.

### Data collection tools

#### Part I: Structured interview questionnaire

 It was designed by the researcher to assess demographic characteristics and patient’s substance abuse history among patients with substance use disorders including as follows:

1. Demographic characteristics of patients with substance use disorders: it included age, marital status, educational level, occupation, average monthly income, and home participants.
2. Patient’s substance abuse history: it included age at the beginning of substance abuse, number of substances, method of substance use, duration of substance abuse, number of hospital admission, suffering from chronic diseases, experiencing health complications due to overdose, family history for substance abuse, the number of relapse, reasons of relapses, and previous enrollment in rehabilitative program.

- Years of use was defined as the self-reported total duration (in years) of regular substance use since the age of onset. Clinical variables (e.g., method of drug use, chronic disease status, previous enrollment in rehabilitation programs) were collected using a structured questionnaire developed by the authors.

#### Part II: Stimulant Relapse Risk Scale (SRRS)

 It was developed by Ogai et al. [21] and was adopted by the researcher to measure the risk of relapse in individuals with a history of drug dependence. Higher scores indicating a greater risk of relapse. The scale consists of validated 35 items across six subscales: anxiety, emotionality, compulsivity, positive expectancies, lack of negative expectancy, and illness insight. Responses were rated on a 3-point Likert scale (1 = Disagree, 2 = Neutral, and 3 = Agree). The scale showed acceptable to excellent internal consistency: Cronbach’s α ranged from 0.62 to 0.90 across the six subscales and 0.86 for the total scale. Factor analysis using five factors found that item loadings in the initial solution ranged from about 0.30 to 0.68; after varimax rotation, loadings strengthened to roughly 0.31–0.74. These five factors together explained about 30% of the total variance. The Kaiser–Meyer–Olkin (KMO) measure was **≈** 0.81, and Bartlett’s test of sphericity was highly significant (*p* ≤ 0.001), indicating that the data were suitable for factor analysis. All items on the scale were retained.

#### Part III: Frustration Discomfort Scale (FDS)

 It was developed by Harrington [22] and was adopted by the researcher to investigate participants’ frustration tolerance and their ability to cope with frustrating situations and discomfort. The scale consisted of 28 items across four domains: emotional intolerance, entitlement, discomfort intolerance, and achievement. Higher scores indicating lower frustration tolerance. Respondents rated the intensity of their distress or frustration in specific situation using a 5-point Likert scale (1 = absent, 2 = mild, 3 = moderate, 4 = strong, and 5 = very strong). The scale demonstrated high reliability, with Cronbach’s α coefficients ranging from 0.85 to 0.90 across its four subscales and 0.95 for the overall scale. In the exploratory factor analysis, initial factor loadings spanned roughly 0.33–0.55, and after varimax rotation they increased to approximately 0.33–0.94. The four rotated factors accounted for **≈** 81.9% of the variance, reflecting a strong factor structure. The KMO measure was **≈** 0.88, and Bartlett’s test was highly significant (*p* ≤ 0.001), confirming the adequacy of the data for factor analysis. No items were removed.

#### Part Ⅳ: Stages of Change Readiness and Treatment Eagerness Scale (SOCRATES)

It was developed by Miller and Tonigan [23] and was adopted by the researcher to assess participants’ motivation and readiness for change in relation to substance use disorders. grounded in the Transtheoretical Model of Change [24]. The scale consisted of 19 items with no reversed scores that yield three subscales: Recognition (Re) corresponds to the pre-contemplation and preparation stage, Ambivalence (Am) corresponds to the contemplation stage and Taking steps (Ts) corresponds to the action and maintenance stage. Higher scores reflect greater recognition, less ambivalence, and taking more steps towards change. Responses were rated on a five-point Likert scale (1 = strongly disagree, 2 = disagree, 3 = unsure, 4 = agree, and 5 = strongly agree). The scale showed excellent internal consistency, with Cronbach’s α values ranged from 0.84 to 0.93 across the three subscales (recognition, ambivalence and taking steps) and 0.94 for the total scale, indicating excellent reliability. Exploratory factor analysis yielded factor loadings between 0.32 and 0.56 in the unrotated solution. Varimax rotation improved these loadings to **≈** 0.32–0.83, and the three factors together explained about 70.7**%** of the variance. The KMO statistic was **≈** 0.90, and Bartlett’s test was again highly significant (*p* ≤ 0.001), demonstrating that the correlation matrix was appropriate for factor analysis. All items were retained.

### Tool preparation & pilot study

The research instruments were translated into Arabic by bilingual experts fluent in both English and Arabic. Accuracy and cultural relevance were emphasized throughout the translation process. To validate these translations, each was rigorously back-translated into English to ensure linguistic equivalence and to identify any discrepancies. Following this, face-validity assessments were conducted for each instrument, with panels of experts meticulously reviewing the Arabic versions to confirm that they captured the intended constructs accurately within the Arabic context. Feedback was also gathered from potential participants to evaluate the clarity, relevance and cultural appropriateness of the translated items. Reliability was assessed using statistical methods specifically Cronbach’s alpha to ensure adequate internal consistency. A pilot study involving 29 participants was then conducted to test the translated instruments’ clarity, relevance and reliability. These participants were subsequently excluded from the main study. The results of the pilot study indicated that no further modifications to the instruments were necessary.

### Data collection procedure

Data collection was conducted over six months, from May to October 2024. Patients were approached during clinic visits and briefed about the study. Each participant was individually interviewed by the researchers to collect the required data using the study tools on their scheduled follow-up day. Interviews were conducted in a quiet, well-ventilated area, and each session lasted approximately 30–45 min.

### Ethical considerations

The study was approved by the Ethics Committee of the Faculty of Nursing at Ain Shams University, Cairo, Egypt (Approval Code: 24.12.445). All procedures adhered to the ethical standards outlined in the Declaration of Helsinki. Prior to completing the questionnaire, written informed consent was obtained from each participant. The researchers assured participants of the anonymity and confidentiality of their responses and explained the purpose of the study. Participants were informed of their right to refuse participation without any impact on their care. They were also informed that they could withdraw from the study at any time, even after it had begun, and that their privacy and confidentiality would be strictly maintained.

### Statistical analysis

Data were analyzed using IBM SPSS Statistics version 27. Descriptive statistics included frequencies and percentages for categorical variables and means with standard deviations (SD) for continuous variables. Chi-square tests were employed to examine associations between categorical socio-demographic variables (e.g., marital status, educational level) and outcomes (relapse risk, frustration tolerance, motivational readiness). Pearson correlations quantified relationships between continuous psychological constructs (relapse risk, frustration tolerance, motivational readiness), with significance set at *p* ≤ 0.05. Effect sizes were interpreted as follows: *r* = 0.10 (small), 0.30 (moderate), and 0.50 (large) [25]. Key analyses focused on three primary constructs: relapse risk (stratified into low, moderate, and high categories using SRRS domain scores), frustration tolerance (assessed via total and subdomain scores, including emotional intolerance and entitlement), and motivational readiness (evaluated through subscales: recognition, ambivalence, and taking steps). Relations between these variables were examined using Pearson correlations. Chi-square tests identified significant socio-demographic associations with relapse risk and frustration tolerance (*p* < 0.01). All analyses adhered to assumptions of independence and linearity, with results reported at 95% confidence intervals.

## Results

Table 1 clarifies that 44.4% of the studied patients with substance use disorders were aged between 20 and 30 years old, with a mean age of 31.76 ± 7.99. Also, 44.8% of them were single and 36.5% finished secondary education. Additionally, 46.5% of them were employed. As regard Adequacy of monthly income, 48.3% of them had insufficient monthly income. Regarding their home participants, 52.8% of them lived with their relatives.

**Table 1:** Frequency distribution of the studied patients with substance use disorders according to their demographic data (*n* = 288)

Items	No.	%
Age (years)		
15 < 20	14	4.9
20 < 30	128	44.4
30 < 40	108	37.5
40 < 50	34	11.8
≥ 50	4	1.4
Mean ± SD31.76 ± 7.99		
Marital status		
Single	129	44.8
Married	108	37.5
Divorced	41	14.2
Widowed	10	3.5
Educational level		
Illiterate	36	12.5
Primary education	80	27.8
Secondary education	105	36.5
Higher education	67	23.2
Occupation		
No work	90	31.3
Student	34	11.8
Employee	134	46.5
Housewife	30	10.4
Average monthly income		
Not enough	139	48.3
Enough	38	13.2
Barely enough	111	38.5
Home participants		
Alone	42	14.6
With spouse	94	32.6
With relatives	152	52.8

Table 2 shows that 42.4% of the studied patients with substance use disorders whose starting age of onset of substance abuse was between 20 and 30 years with mean 24.43 ± 7.21. Also, 76.4% of them used 2 to 4 substances. In addition, 79.5% of them abused opioids and hallucinogens as the most common substances. Regarding the primary method of substance use, 49.3% of them combined different methods and 78.5% of them had years of substance use from 1 to 10 years with mean 7.16 ± 5.81, and 78.1% of them admitted for hospital from 1 to 3 times with mean 2.59 ± 1.65. Also, 89.6% of them reported no chronic diseases and 80.6% of them reported no health complications due to overdose. As regard family history for substance abuse, 54.9% of them had family history of substance abused. Regarding the number of patients relapses, 68.1% of them experienced relapses at least from 1 to 3 times. Lastly, 66.3% had not previously enrolled in a rehabilitation program.

**Table 2:** Frequency distribution of the studied patients with substance use disorders according to their substance abuse history (*n* = 288)

Items	NO.		%
Age of onset of substance use			
12–20	115		39.9
21–30	122		42.4
31–40	49		17
> 40	2		0.7
Mean ± SD24.43 ± 7.21			
Number of substances			
One substance	36		12.5
2–4	220		76.4
≥ 5	32		11.1
Mean ± SD 2.87 ± 1.27			
Types of abused substances*			
Opioids	229		79.5
CNS Depressants	168		58.3
CNS Stimulants	52		18.1
Hallucinogens	229		79.5
Inhalants	21		7.3
Synthetic drugs	122		42.4
Method of drug use			
Oral	104		36.1
Nasal	24		8.3
Injection	18		6.3
Combinations of the above	142		49.3
Years of use			
1–10	226		78.5
11–20	54		18.7
21–30	8		2.8
Mean ± SD7.16 ± 5.81			
Number of inpatient treatment episodes (Hospital Admission)			
1–3	225		78.1
4–6	53		18.4
7–10	10		3.5
Mean ± SD2.59 ± 1.65			
Suffer from any chronic diseases			
Yes	30		10.4
No	258		89.6
Experiencing health complications due to overdose			
Yes	56		19.4
No	232		80.6
Family member using drugs			
Yes	130		45.1
No	158		54.9
The number of relapses the patient experienced			
No relapse	35		12.2
1–3	196		68.1
4–6	47		16.3
7–10	10		3.5
Mean ± SD2.23 ± 1.81			
Previous enrollment in a rehabilitative program			
Yes		97	33.7
No		191	66.3

*The numbers are not mutually exclusive

The results provide a comprehensive analysis of relapse risk, frustration tolerance, and motivational readiness for change among patients with SUD across various subdomains, highlighting critical areas of concern. High emotionality problems 62.8% and lack of negative expectancy for drugs 64.2% were notably prevalent among high-risk individuals, whereas compulsivity for drugs was most frequent in the low-risk group 63.9%, reflecting varied patterns of vulnerability. Similarly, frustration tolerance emerged as a significant issue, with high levels of emotional intolerance 61.5%, entitlement 61.8%, and discomfort intolerance 59%, and achievement being the most severely impacted domain 64.6%. Motivational readiness for change revealed that a majority of patients in the low-risk category demonstrated recognition of their substance use issues 72.9%, while ambivalence was a significant barrier for 49.3% of high-risk individuals, indicating considerable internal conflict in their readiness to commit to change, see more in Table 3.

**Table 3:** Distribution of studied patients with substance use disorders according to their levels of total relapse risk, frustration tolerance and motivational readiness for change (*n* = 288)

	Low		Moderate		High	
	*N*	%	*N*	%	*N*	%
Relapse Risk						
Anxiety and intention to use drug (AI)	133	46.2	71	24.6	84	29.2
Emotionality problems (EP)	36	12.5	71	24.7	181	62.8
Compulsivity for drug (CD)	184	63.9	30	10.4	74	25.7
Positive expectancies and lack of control over drug (PL)	85	29.5	70	24.3	133	46.2
Lack of negative expectancy for the drug (NE)	32	11.1	71	24.7	185	64.2
Insight into illness	49	17	111	38.5	128	44.5
Total	75	26	125	43.4	88	30.6
Frustration Tolerance						
Discomfort Intolerance	50	17.4	68	23.6	170	59
Entitlement	64	22.2	46	16	178	61.8
Emotional Intolerance	42	14.5	69	24	177	61.5
Achievement	52	18	50	17.4	186	64.6
Total	50	17.4	68	23.6	170	59
Motivational Readiness for Change						
Recognition (Re)	210	72.9	40	13.9	38	13.2
Ambivalence (Am)	68	23.6	78	27.1	142	49.3
Taking Steps (Ts)	122	42.4	48	16.6	118	41
Total	138	48	63	22	87	30

Table 4 displays that there were highly statistically significant relations between total level of relapse risk and demographic data of the studied patients in the form of marital status, educational level, occupation, and home participants in which X²= 28.582, 34.750, 18.024, and 17.935 at (*P* < 0.01). While there were no statistically significant relations with their age, and average monthly income in which X²= 9.540, and 4.403. at (*P* > 0.05).

**Table 4:** Relation between socio-demographic data and levels of total relapse risk among the studied patients with substance use disorders (*n* = 288)

Patients’ demographic data	Total levels of relapse risk						X^2^	*P* value
	Low		Moderate		High			
	No.	%	No.	%	No.	%		
Age (years)								
15 < 20	2	0.7	8	2.8	4	1.4	9.540	0.299
20 < 30	26	9.0	58	20.1	44	15.3		
30 < 40	34	11.8	41	14.2	33	11.5		
40 < 50	11	3.8	16	5.6	7	2.4		
≥ 50	2	0.7	2	0.7	0	0.0		
Marital status								
Single	22	7.6	68	23.6	39	13.5	28.582	0.000**
Married	29	10.1	36	12.5	43	14.9		
Divorced	20	6.9	15	5.2	6	2.1		
Widowed	4	1.4	6	2.1	0	0.0		
Educational level								
Illiterate	22	7.6	12	4.2	2	0.7	34.750	0.000**
Primary education	18	6.3	30	10.4	32	11.1		
Secondary education	22	7.6	55	19.1	28	9.7		
Higher education	13	4.5	28	9.7	26	9.0		
Occupation								
No work	37	12.8	35	12.2	18	6.3	18.024	0.006**
Student	6	2.1	16	5.6	12	4.2		
Employee	28	9.7	58	20.1	48	16.7		
Housewife	4	1.4	16	5.6	10	3.5		
Average monthly income								
Not enough	34	11.8	55	19.1	50	17.4	4.403	0.354
Enough	12	4.2	18	6.3	8	2.8		
Barely enough	29	10.1	52	18.1	30	10.4		
Home participants								
Alone	8	2.8	24	8.3	10	3.5	17.935	0.001**
With spouse	21	7.3	30	10.4	43	14.9		
With relatives	46	16.0	71	24.7	35	12.2		

**highly significant at *p* ‹ 0.01

Table 5 displays that there were highly statistically significant relations between total level of relapse risk and substance abuse history of the studied patients in the form of method of substance use, suffering from chronic diseases, number of relapses and previous enrollment in a rehabilitative program in which X²= 36.668, 28.700, 22.973 and 21.044 at (*P* < 0.01). Also, there were statistically significant relations with their age of onset of substance abuse, and number of hospital admission in which X²= 13.505, and 12.578 at (*P* < 0.05). While there were no statistically significant relations with their years of substance use, number of substances, experiencing health complications due to overdose, and family history of substance abuse in which X²= 3.549, 4.136, 3.950, and 3.020 at (*P* > 0.05).

**Table 5:** Relation between substance abuse history of the studied patients with substance use disorders and their total relapse risk levels (*n* = 288)

Substance abuse history	Total levels of relapse risk						X^2^	*P* value
	Low		Moderate		High			
	No.	%	No	%	No.	%		
Age of onset of substance abuse								
12 < 20	28	9.7	42	14.6	45	15.6	13.505	0.036*
20 < 30	30	10.4	59	20.5	33	11.5		
30 < 40	15	5.2	24	8.3	10	3.5		
≥ 40	2	0.7	0	0.0	0	0.0		
Years of use								
1–10	59	20.5	99	34.4	68	23.6	3.549	0.471
11–20	16	5.6	22	7.6	16	5.6		
21–30	0	0.0	4	1.4	4	1.4		
Number of substances								
One substance	10	3.5	16	5.6	10	3.5	4.136	0.388
2–4	56	19.4	100	34.7	64	22.2		
≥ 5	9	3.1	9	3.1	14	4.9		
Method of substance use								
Oral	38	13.2	30	10.4	36	12.5	36.668	0.000**
Nasal	4	1.4	18	6.3	2	0.7		
Injection	0	0.0	16	5.6	2	0.7		
Combinations of the above	33	11.5	61	21.2	48	16.7		
Number of inpatient treatment episodes (Hospital Admission)								
1–3	56	19.4	107	37.2	62	21.5	12.578	0.014*
4–6	17	5.9	12	4.2	24	8.3		
7–10	2	0.7	6	2.1	2	0.7		
Suffering from any chronic diseases								
Yes	20	6.9	6	2.1	4	1.4	28.700	0.000**
No	55	19.1	119	41.3	84	29.2		
Experiencing health complications due to overdose								
Yes	19	6.6	18	6.3	19	6.6	3.950	0.139
No	56	19.4	107	37.2	69	24.0		
Family history of substance abuse								
Yes	38	13.2	65	22.6	55	19.1	3.020	0.221
No	37	12.8	60	20.8	33	11.5		
The number of relapses the patient experienced								
No relapse	6	2.1	22	7.6	7	2.4	22.973	< 0.001**
1–3	52	18.1	89	30.9	55	19.1		
4–6	17	5.9	8	2.8	22	7.6		
7–10	0	0.0	6	2.1	4	1.4		
Previous enrollment in a rehabilitative program								
Yes	23	8.0	28	9.7	46	16.0	21.044	0.000**
No	52	18.1	97	33.7	42	14.6		

*Significant at *p* ‹ 0.05. **highly significant at *p* ‹ 0.01

Table 6 shows that there were highly statistically significant relations between total level of frustration tolerance and demographic data of the studied patients in the form of age, educational level, and occupation in which X²= 24.253, 37.220, and 26.204 at (*P* < 0.01). Also, there were statistically significant relations with their average monthly income in which X²= 9.891 at (*P* < 0.05). While there were no statistically significant relations with their marital status, and home participants in which X²= 8.047, and 8.585 at (*P* > 0.05).

**Table 6:** Relation between demographic data of the studied patients with substance use disorders and their levels of total frustration tolerance (*n*=288)

Patients’ demographic data	Total levels of frustration tolerance						X^2^	*P* value
	Low		Moderate		High			
	No.	%	No	%	No	%		
Age (years)								
15 < 20	2	0.7	4	1.4	8	2.8	24.253	0.002**
20 < 30	30	10.4	22	7.6	76	26.4		
30 < 40	10	3.5	30	10.4	68	23.6		
40 < 50	8	2.8	8	2.8	18	6.3		
≥ 50	0	0.0	4	1.4	0	0.0		
Marital status								
Single	26	9.0	26	9.0	77	26.7	8.047	0.235
Married	16	5.6	24	8.3	68	23.6		
Divorced	8	2.8	14	4.9	19	6.6		
Widowed	0	0.0	4	1.4	6	2.1		
Educational level								
Illiterate	12	4.2	14	4.9	10	3.5	37.220	0.000**
Primary education	16	5.6	24	8.3	40	13.9		
Secondary education	16	5.6	26	9.0	63	21.9		
Higher education	6	2.1	4	1.4	57	19.8		
Occupation								
No work	30	10.4	18	6.3	42	14.6	26.204	0.000**
Student	4	1.4	6	2.1	24	8.3		
Employee	14	4.9	38	13.2	82	28.5		
Housewife	2	0.7	6	2.1	22	7.6		
Average monthly income								
Not enough	20	6.9	32	11.1	87	30.2	9.891	0.042*
Enough	12	4.2	4	1.4	22	7.6		
Barely enough	18	6.3	32	11.1	61	21.2		
Home participants								
Alone	10	3.5	12	4.2	20	6.9	8.585	0.072
With spouse	10	3.5	18	6.3	66	22.9		
With relatives	30	10.4	38	13.2	84	29.2		

*Significant at *p* ‹ 0.05. **highly significant at *p* ‹ 0.01

Table 7 shows that there were highly statistically significant relations between total level of frustration tolerance and substance abuse history of the studied patients in the form of age of onset of substance abuse, years of substance use, method of substance use, suffering from chronic diseases, and previous enrollment in a rehabilitative program in which X²= 27.515, 15.830, 46.030, 14.672, and 15.187 at (*P* < 0.01). Also, there were statistically significant relations with their number of substances and experiencing health complications due to overdose in which X²= 10.093, and 6.132 at (*P* < 0.05). While there were no statistically significant relations with their number of hospital admission, family history of substance abuse, and number of relapses in which X²= 4.908,0.815, and 10.649 at (*P* > 0.05).

**Table 7:** Relation between substance abuse history of the studied patients with substance use disorders and their levels of total frustration tolerance (*n* = 288)

Substance abuse history	Total levels of frustration tolerance						X^2^	*P* value
	Low		Moderate		High			
	No.	%	No	%	No	%		
Age of onset of substance abuse								
12 < 20	30	10.4	26	9.0	59	20.5	27.515	0.000**
20 < 30	20	6.9	22	7.6	80	27.8		
30 < 40	0	0.0	18	6.3	31	10.8		
≥ 40	0	0.0	2	0.7	0	0.0		
Years of use								
1–10	32	11.1	48	16.7	146	50.7	15.830	0.003**
11–20	16	5.6	16	5.6	22	7.6		
21–30	2	0.7	4	1.4	2	0.7		
Number of substances								
One substance	10	3.5	10	3.5	16	5.6	10.093	0.039*
2–4	34	11.8	56	19.4	130	45.1		
≥ 5	6	2.1	2	0.7	24	8.3		
Method of substance use								
Oral	24	8.3	24	8.3	56	19.4	46.030	0.000**
Nasal	14	4.9	6	2.1	4	1.4		
Injection	2	0.7	6	2.1	10	3.5		
Combinations of the above	10	3.5	32	11.1	100	34.7		
Number of inpatient treatment episodes (Hospital admission)								
1–3	40	13.9	56	19.4	129	44.8	4.908	0.297
4–6	8	2.8	8	2.8	37	12.8		
7–10	2	0.7	4	1.4	4	1.4		
Suffering from any chronic diseases								
Yes	10	3.5	12	4.2	8	2.8	14.672	0.001**
No	40	13.9	56	19.4	162	56.3		
Experiencing health complications due to overdose								
Yes	16	5.6	12	4.2	28	9.7	6.132	0.04*
No	34	11.8	56	19.4	142	49.3		
Family history of substance abuse								
Yes	30	10.4	38	13.2	90	31.3	0.815	0.665
No	20	6.9	30	10.4	80	27.8		
The number of relapses the patient experienced								
No relapse	6	2.1	10	3.5	19	6.6	10.649	0.100
1–3	38	13.2	48	16.7	110	38.2		
4–6	4	1.4	6	2.1	37	12.8		
7–10	2	0.7	4	1.4	4	1.4		
Previous enrollment in a rehabilitative program								
Yes	12	4.2	36	12.5	49	17.0	15.187	0.001**
No	38	13.2	32	11.1	121	42.0		

*Significant at *p* ‹ 0.05. **highly significant at *p* ‹ 0.01

Table 8 displays that there were highly statistically significant relations between total level of motivational readiness for change and demographic data of studied patients in the form of marital status, educational level, occupation, and home participants in which X^2^ = 19.046, 29.631, 38.393, and 15.376 at (*P* < 0.01). While there were no statistically significant relations with their age, and average monthly income in which X^2^ = 14.418, and 6.019 at (*P* > 0.05).

**Table 8:** Relation between demographic data of the studied patient with substance use disorders and their levels of total motivational readiness for change (*n* = 288)

Patients’ demographic data	Total levels of motivational readiness for change						X^2^	*P* value
	Low		Moderate		High			
	No.	%	No.	%	No.	%		
Age (years)								
15 < 20	8	2.8	0	0.0	6	2.1	14.418	0.072
20 < 30	62	21.5	33	11.5	33	11.5		
30 < 40	44	15.3	25	8.7	39	13.5		
40 < 50	20	6.9	5	1.7	9	3.1		
≥ 50	4	1.4	0	0.0	0	0.0		
Marital status								
Single	72	25.0	28	9.7	29	10.1	19.046	0.004**
Married	42	14.6	27	9.4	39	13.5		
Divorced	18	6.3	4	1.4	19	6.6		
Widowed	6	2.1	4	1.4	0	0.0		
Educational level								
Illiterate	22	7.6	4	1.4	10	3.5	29.631	0.000**
Primary education	40	13.9	20	6.9	20	6.9		
Secondary education	62	21.5	16	5.6	27	9.4		
Higher education	14	4.9	23	8.0	30	10.4		
Occupation								
No work	54	18.8	12	4.2	24	8.3	38.393	0.000**
Student	16	5.6	11	3.8	7	2.4		
Employee	64	22.2	36	12.5	34	11.8		
Housewife	4	1.4	4	1.4	22	7.6		
Average monthly income								
Not enough	60	20.8	33	11.5	46	16.0	6.019	0.198
Enough	24	8.3	8	2.8	6	2.1		
Barely enough	54	18.8	22	7.6	35	12.2		
Home participants								
Alone	30	10.4	6	2.1	6	2.1	15.376	0.004**
With spouse	34	11.8	27	9.4	33	11.5		
With relatives	74	25.7	30	10.4	48	16.7		

**highly significant at *p* ‹ 0.01

Table 9 displays that there were highly statistically significant relations between total level of motivational readiness for change and substance abuse history of the studied patients in the form of number of substances, method of substance use, number of hospital admission, family history of substance abuse, and number of relapses in which X²= 28.800, 49.471, 14.065, 10.105, and 35.103 at (*P* < 0.01). Also, there were statistically significant relations with their years of substance use, suffering from chronic diseases, and previous enrollment in a rehabilitative program in which X²= 11.980, 6.137, and 7.723 at (*P* < 0.05). While there were no statistically significant relations with their age of onset of substance abuse and experiencing health complications due to overdose in which X²= 9.748, and 4.918 at (*P* > 0.05).

**Table 9:** Relation between substance abuse history of the studied patient with substance use disorders and their levels of total motivational readiness for change (*n* = 288)

Substance abuse history	Total levels of motivational readiness for change						X^2^	*P* value
	Low		Moderate		High			
	No.	%	No.	%	No.	%		
Age of onset of substance abuse								
12 < 20	64	22.2	24	8.3	27	9.4	9.748	0.136
20 < 30	50	17.4	31	10.8	41	14.2		
30 < 40	22	7.6	8	2.8	19	6.6		
≥ 40	2	0.7	0	0.0	0	0.0		
Years of use								
1–10	98	34.0	51	17.7	77	26.7	11.980	0.017*
11–20	34	11.8	12	4.2	8	2.8		
21–30	6	2.1	0	0.0	2	0.7		
Number of substances								
One substance	26	9.0	8	2.8	2	0.7	28.800	< 0.001**
2–4	108	37.5	45	15.6	67	23.3		
≥ 5	4	1.4	10	3.5	18	6.3		
Method of substance use								
Oral	62	21.5	21	7.3	21	7.3	49.471	0.000**
Nasal	16	5.6	2	0.7	6	2.1		
Injection	18	6.3	0	0.0	0	0.0		
Combinations of the above	42	14.6	40	13.9	60	20.8		
Number of inpatient treatment episodes (Hospital Admission)								
1–3	120	41.7	47	16.3	58	20.1	14.065	0.007**
4–6	14	4.9	14	4.9	25	8.7		
7–10	4	1.4	2	0.7	4	1.4		
Suffering from any chronic diseases								
Yes	20	6.9	2	0.7	8	2.8	6.137	0.046*
No	118	41.0	61	21.2	79	27.4		
Experiencing health complications due to overdose								
Yes	28	9.7	17	5.9	11	3.8	4.918	0.086
No	110	38.2	46	16.0	76	26.4		
Family history of substance abuse								
Yes	76	26.4	44	15.3	38	13.2	10.105	0.006**
No	62	21.5	19	6.6	49	17.0		
The number of relapses the patient experienced								
No relapse	26	9.0	4	1.4	5	1.7	35.103	< 0.001**
1–3	102	35.4	43	14.9	51	17.7		
4–6	8	2.8	14	4.9	25	8.7		
7–10	2	0.7	2	0.7	6	2.1		
Previous enrollment in a rehabilitative program								
Yes	44	15.3	30	10.4	23	8.0	7.723	0.021*
No	94	32.6	33	11.5	64	22.2		

*Significant at *p* ‹ 0.05. **highly significant at *p* ‹ 0.01

**Fig. 1:**
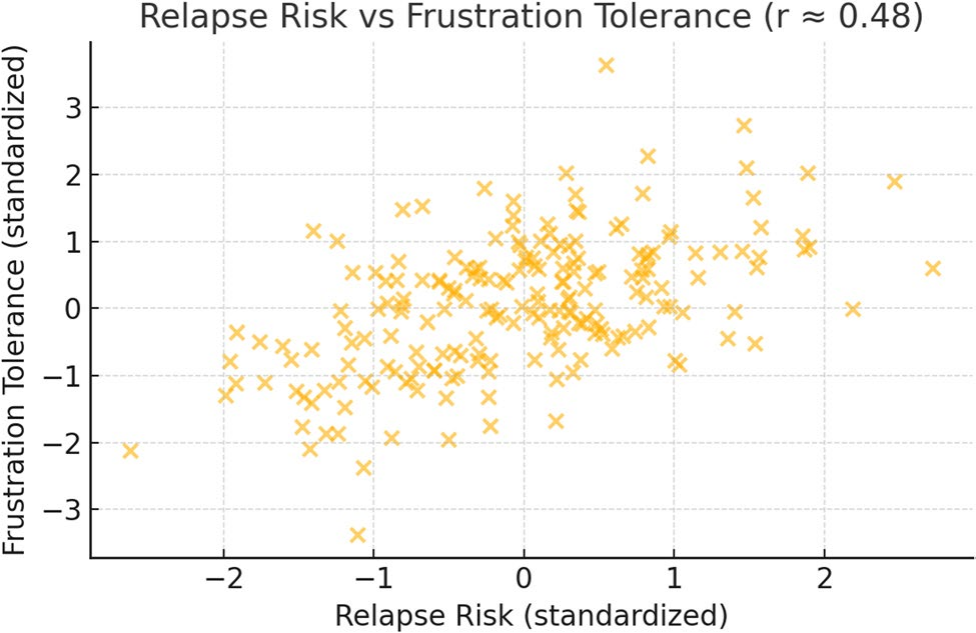
Association Between Relapse Risk and Frustration Tolerance in Patients With Substance Use Disorders

Figure 1 illustrates the positive association between relapse risk and frustration tolerance among patients with substance use disorders. The moderate-to-strong correlation (*r* ≈ 0.48) suggests that individuals with higher relapse risk also report significantly lower tolerance for frustration.

**Fig. 2:**
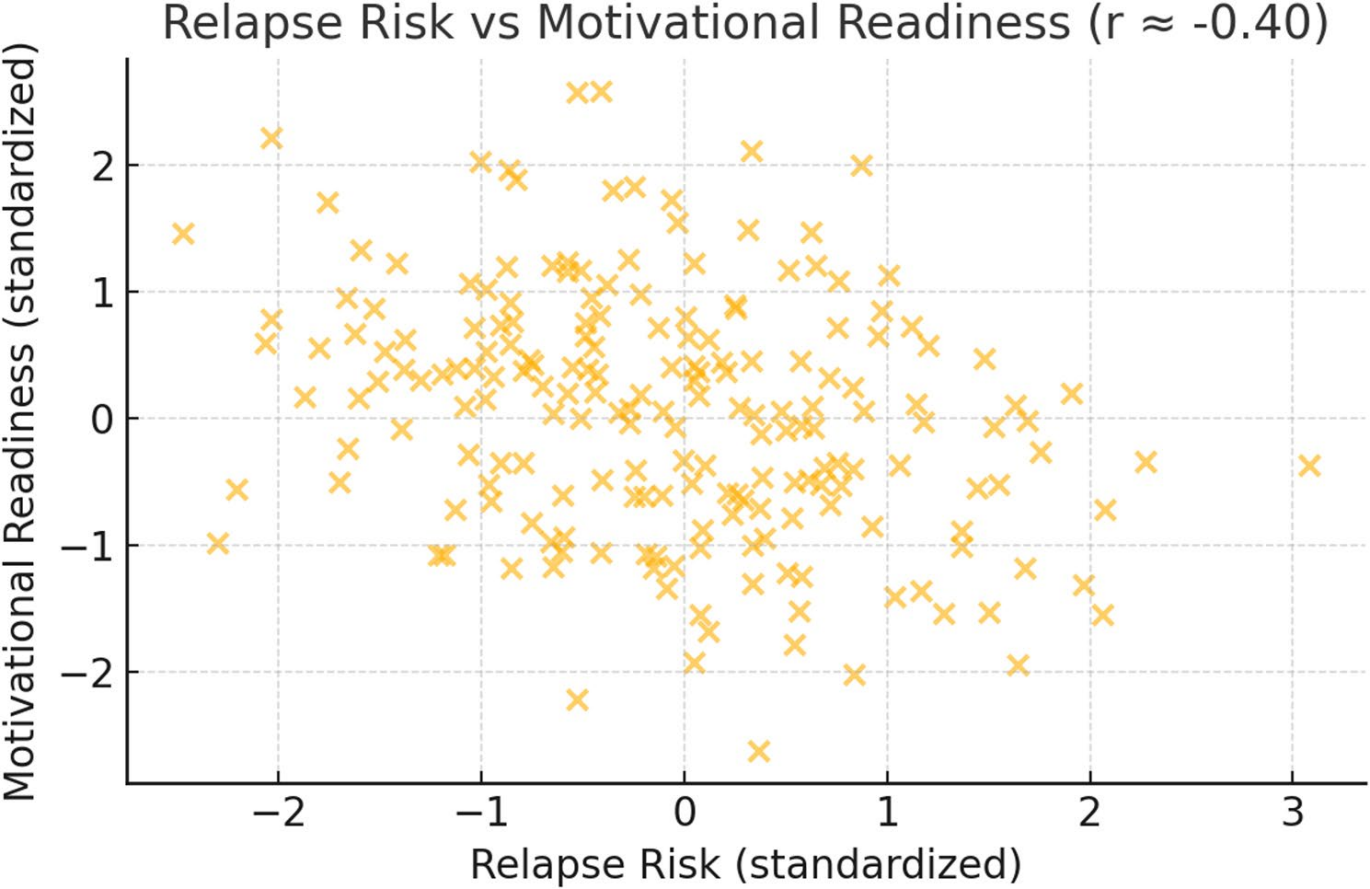
Negative Correlation Between Relapse Risk and Motivational Readiness for Change

Figure 2 shows the negative correlation between relapse risk and motivational readiness for change (*r* ≈ − 0.40). Patients exhibiting greater risk of relapse tend to demonstrate reduced readiness to change, indicating that ambivalence and motivational barriers may coexist with heightened vulnerability.

**Fig. 3:**
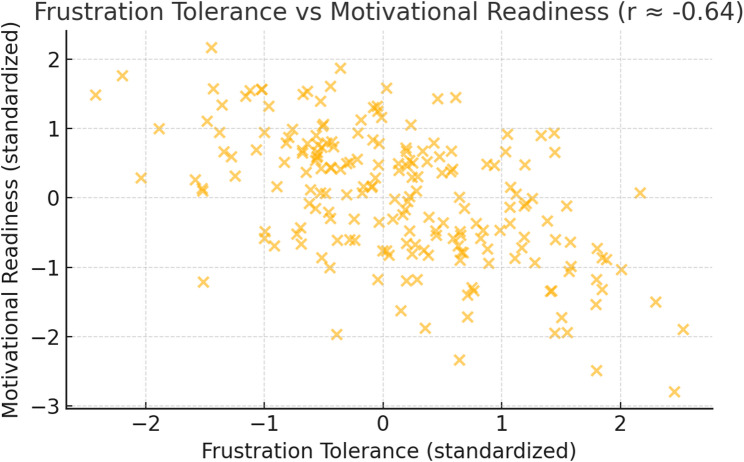
Inverse Relationship Between Frustration Tolerance and Motivational Readiness for Change

This scatterplot in Fig. 3 depicts the strong negative relationship between frustration tolerance and motivational readiness (*r* ≈ − 0.64). Patients with low tolerance for frustration are more likely to exhibit diminished readiness to engage in behavioral change, suggesting a synergistic effect of emotional dysregulation and motivational deficits on treatment engagement.

Table 10 reveals that there was a moderate negative correlation between the relapse risk of the studied patients and their motivational readiness for change in which (*r* = − 0.399, 95% CI [–0.49, − 0.30], *p* < 0.001). Also, there was a strong negative correlation between frustration intolerance of the studied patients and their motivational readiness for change in which (*r* = − 0.643, 95% CI [–0.71, − 0.57], *p* < 0.001). While there was a moderate positive correlation between the relapse risk and their frustration discomfort and tolerance among the studied patients in which (*r* = 0.482, 95% CI [0.39, 0.57], *p* < 0.001).

**Table 10:** Correlation between relapse risk, frustration intolerance and motivational readiness for change among the studied patients with substance use disorders (*n* = 288)

Items	*r*	95% CI	*p*-value
Relapse risk - Frustration intolerance	0.482**	[0.39, 0.57]	< 0.001
Relapse risk – Motivational readiness	− 0.399**	[–0.49, − 0.30]	< 0.001
Frustration intolerance – Motivational readiness	− 0.643**	[–0.71, − 0.57]	< 0.001

(**) Statistically significant at *p*<0.01. r Pearson correlation

## Discussion

This study provides one of the first systematic examinations of relapse risk, frustration tolerance, and motivational readiness for change among Egyptian outpatients with substance use disorders (SUDs). By situating our findings within both established theoretical frameworks and the unique sociocultural context of Egypt, we highlight several contributions to the understanding of relapse vulnerability.

### Relapse risk and frustration tolerance

Patients with higher relapse risk exhibited greater emotional dysregulation and a diminished sense of negative expectancies regarding drug use. These results mirror findings from Western contexts by Hand et al. [14] and Byllesby et al. [26], where poor emotional regulation consistently predicts relapse. Similarly, Michalczuk et al. [27], who reported that diminished frustration tolerance predicted impulsive behavior and relapse in SUD patients. However, our study extend this evidence by showing that frustration intolerance was especially pronounced in domains of entitlement and achievement dimensions that may be exacerbated in Egypt by high youth unemployment and unmet social expectations. Also, this result agreed with a recent meta-analysis of 22 studies involving 1936 individuals with SUDs showed that people with addictions have significantly higher difficulties in emotion regulation compared with healthy controls [28].

Our results further reveal a strong positive correlation between relapse risk and frustration intolerance. This supports theoretical models positing that low distress tolerance impairs coping and triggers impulsive substance use [29]. These findings are consistent with Baars et al. [30], who indicates that individuals whose distress tolerance fails to improve during treatment are more likely to relapse, and with Harrington [22], work identifying frustration intolerance dimensions associated with psychological distress and impaired recovery. Similarly, a study performed by Anderson et al. [31], who conducted a study across seven countries found that distress tolerance is inversely related to alcohol and cannabis problems, and that negative reinforcement motives using substances to cope with unpleasant emotions mediate this relationship.

This recent result diary-card study of dialectical behavior therapy was in harmony with McCool et al. [32], found that emotion-regulation and mindfulness skills were related to decreased urges to use alcohol or other substances, and that previous-day distress-tolerance skills predicted lower urges among people who initially used substances frequently.

### Relapse risk and motivational readiness

Motivational readiness for change showed a moderate inverse relationship with relapse risk. Participants with greater recognition of their problem and higher commitment to change displayed lower relapse risk, consistent with the Transtheoretical Model and findings that motivational interviewing and enhanced readiness predict better outcomes [33] and prior studies [34, 35] reported that improvements in motivation mediate reductions in substance use. Patients who recognized the severity of their condition and actively engaged in change efforts were less likely to be at high relapse risk. Yet, nearly half of high-risk patients displayed ambivalence, underscoring motivational conflict.

In the Egyptian context, ambivalence may reflect competing pressures: strong family expectations to recover on the one hand, and stigma, shame, and limited rehabilitation opportunities on the other. This result was parallel with Ajiboye [36], who found that patients with higher readiness for change were less likely to experience relapse, as evidenced by a significant negative correlation between these variables. The study further underscored ambivalence, or a lack of motivation, as a strong predictor of relapse, highlighting the importance of enhancing patients’ motivation before initiating treatment.

In the same field, the only previous Egyptian intervention study conducted by Abdel Moneam et al. [37], who combining motivational interviewing and cognitive-behavioral therapy found that participants receiving the integrated intervention had significantly fewer days of drug use and longer time to relapse compared with those attending only 12-step meetings demonstrating that bolstering distress tolerance and emotion-regulation skills through mindfulness practice can further enhance outcomes.

Similarly, Yazıcı et al. [10], who mentioned that a significant negative correlation between relapse risk and readiness to change in patients with SUDs. Additionally, therapists should focus on enhancing motivational readiness and emotional regulation skills to reduce relapse rates.

### Frustration tolerance and motivational readiness

The observed associations underscore the interplay between motivation and emotional regulation in SUD recovery. Relapse risk correlated positively with frustration intolerance, while motivation was inversely related to both variables. These patterns align with evidence from Yamashita and Yoshioka [38] and others [39, 40] highlighting the combined influence of psychological and sociological factors in relapse vulnerability; our results confirm its presence in Egypt while also suggesting that socio-economic stressors intensify its impact. Patients with lower frustration tolerance appeared less willing or able to commit to change, possibly because daily financial stressors, unemployment, and unstable housing further erode their capacity to endure discomfort.

These findings matched with a study conducted by Lin et al. [41], who found no significant association between frustration discomfort and gender as indicated by the lack of significant interaction between gender and Frustration Discomfort Scale (FDS) scores.

Motivation for change emerged as a critical factor in treatment engagement, consistent with Oji et al. [42] and Fiabane et al. [43]. Higher motivation is linked to greater retention and reduced relapse risk, while low frustration tolerance may exacerbate vulnerability, particularly among individuals sensitive to interpersonal stress [44]. Also, Interventions fostering motivation, coping skills, and structured stage-based strategies may enhance outcomes and support sustained recovery, promoting a more patient-centered approach to care [45].

Moreover, this study was agreed upon and endorsed by de Weert-Van Oene et al. [46], revealed that lower readiness for both change and treatment significantly predicted premature attrition from treatment programs, thereby increasing the risk of relapse.

### Socio-Demographic and contextual factors

Sociodemographic and clinical variables influenced the psychological constructs. Longer duration of substance use and unemployment predicted higher relapse risk, while education and employment emerged as protective factors, correlating with higher motivational readiness and lower relapse risk. However, in our Egyptian sample, family living arrangements also shaped outcomes: patients living with relatives often reported higher ambivalence, possibly reflecting both protective oversight and relational stress.

These associations consistent with research linking socio‑economic instability to poor treatment adherence and relapse. Addressing social determinants of health through vocational training, job placement, educational opportunities and family support may therefore augment the effectiveness of psychological interventions [47, 48].

These findings are consistent with Al-Musway [49] and Petrova et al. [50], who identified social pressures, access to substances, and the absence of supportive networks as relapse drivers, while education and employment promote remission. Social stability thus appears integral to motivation and relapse prevention, complementing psychological interventions. Likewise, a study carried out by Nejad et al. [51], reported that age and gender were not significant predictors of substance use.

### Clinical implications

The interconnectedness of relapse risk, frustration tolerance, and motivational readiness for change highlights the need for multidimensional relapse prevention strategies. Combining cognitive-behavioral approaches to address cognitive distortions, skills training to build frustration tolerance, and motivational enhancement techniques could collectively strengthen recovery trajectories. Socio-demographic factors, such as education and employment, also played a protective role, reinforcing the value of psychosocial support programs that stabilize patients’ economic and social contexts as part of comprehensive care.

### Strengths and limitations

This study has several strengths. It addresses a gap in the literature by examining psychological predictors of relapse in an Egyptian sample using validated instruments, and it contextualizes findings within socio‑cultural factors unique to the region. The relatively large sample size enhances the stability of correlations. Nonetheless, several limitations must be considered. The cross-sectional design precludes causal inferences, and self-report measures are susceptible to recall and social-desirability biases. Recruiting from a single outpatient clinic limits generalizability to other settings or populations. In addition, because the majority of patients reported polysubstance use (most commonly opioids and hallucinogens), it was not feasible to stratify relapse risk by primary substance. Future studies with larger samples stratified by the main substance are warranted to clarify substance-specific relapse patterns.

## Conclusion

This study highlights that emotional dysregulation, low frustration tolerance, and ambivalence about change are prevalent among Egyptian outpatients with substance use disorders and are strongly linked to relapse risk. Our findings reveal that these vulnerabilities are intensified by socio-economic hardship, stigma, and family dynamics unique to the Egyptian context, underscoring the importance of culturally adapted relapse-prevention strategies. By situating psychological constructs within their sociocultural environment, this work contributes novel evidence from a non-Western setting and emphasizes the need for integrated interventions that combine emotional regulation training, motivational enhancement, and social support to sustain long-term recovery.

## Data Availability

The datasets used and/or analyzed during the current study are available from the corresponding author upon reasonable request.

## Abbreviations

SUD: Substance use disorder
SRRS: Stimulant Relapse Risk Scale
FDS: Frustration Discomfort Scale
SOCRATES: Stages of Change Readiness and Treatment Eagerness Scale
